# A historically-controlled Phase III study in adults to characterize the acceptability of a process change for manufacturing inactivated quadrivalent influenza vaccine

**DOI:** 10.1186/1471-2334-14-133

**Published:** 2014-03-10

**Authors:** Varsha K Jain, Vijayalakshmi Chandrasekaran, Long Wang, Ping Li, Aixue Liu, Bruce L Innis

**Affiliations:** 1 GlaxoSmithKline Vaccines, GCDC Non Ops, King of Prussia, PA, USA

**Keywords:** Thimerosal-containing, Quadrivalent, Trivalent, Inactivated, Influenza, Adults

## Abstract

**Background:**

An inactivated quadrivalent influenza vaccine (QIV) was recently licenced in the US as a thimerosal-free formulation presented in a pre-filled syringe. A multidose presentation is preferred in some settings due to reduced acquisition and cold storage costs. We assessed the immunogenicity and safety of a thimerosal-containing QIV formulated using a new manufacturing process for presentation in multidose vials.

**Methods:**

Two Phase III non-randomized studies separately evaluated inactivated trivalent influenza vaccine (TIV; 2010–2011; historical control) and a QIV (2011–2012). The QIV contained the same strains as the TIV plus an additional B strain. Both vaccines contained thimerosal to allow multidose presentation: this preservative was added to the QIV during the final formulation step using a new process, whereas it was added to the TIV early in the manufacturing process using an established method. The TIV study included 50 and 70 subjects aged 18–60 and >60 years, respectively; the QIV study included 56 subjects in each age stratum. Immunogenicity was assessed using hemagglutination-inhibition (HI) assays. Reactogenicity was assessed during the 4-day post-vaccination periods and unsolicited adverse events (AEs) were assessed during the 21-day post-vaccination periods.

**Results:**

The TIV and QIV were immunogenic in both age strata. With the QIV and TIV respectively, the seroconversion rates were 48.2–62.7% and 71.4–83.7% for influenza A, and 33.9–62.5% and 67.3–72.9% for influenza B. With the QIV and TIV respectively, the seroprotection rates were 92.9–98.2% and 98.2–100% for influenza A, and 88.6–100% and 95.9–98.6% for influenza B. Pre-vaccination titers were higher in the QIV versus TIV study which confounds a direct comparison and likely explains the lower seroconversion rates observed in the QIV study. There were no safety concerns raised with TIV or QIV.

**Conclusions:**

The thimerosal-containing QIV formulated using a new process was immunogenic, conforming to regulatory acceptance criteria, with a reactogenicity and safety profile in line with the TIV manufactured using a licensed process. These results support acceptability of a manufacturing process change in which the thimerosal preservative is added at the point at which batches are filled into multidose vials.

**Trial registration:**

These trials were registered at ClinicalTrials.gov: NCT01440387; NCT01153685.

## Background

Influenza is an important cause of outpatient medical visits and worker absenteeism among healthy adults, and elderly adults are particularly vulnerable to influenza-related complications [1-5]. In many countries, annual vaccination of elderly individuals against influenza is recommended to reduce the burden of influenza-related disease. In the US, annual vaccination is recommended for all individuals 6 months of age and older [6].

Seasonal vaccines have been trivalent, containing strains representing influenza A subtypes H1N1 and H3N2, and one influenza B strain, according to recommendations from the World Health Organization (WHO) based on forecasts from their global surveillance program. However, two antigenically distinct influenza B lineages (B/Yamagata and B/Victoria) emerged globally in humans in the early 1980s, and have co-circulated in the US since 2000 [7]. During about half of the influenza seasons in the past decade in the US and Europe, the recommended TIV was mismatched for the predominant circulating influenza B lineage [7,8]. Because there is limited cross-protection between the two influenza B lineages, B-lineage mismatch can reduce vaccine effectiveness [9-11].

The need for a seasonal vaccine that provides protection against both B lineages has prompted the development of quadrivalent vaccines containing a strain from both the B/Yamagata and B/Victoria lineages, and in 2012, the WHO included in its recommendation for the first time four strains for quadrivalent influenza vaccines for use in the vaccine for the 2012–2013 influenza season in the Northern Hemisphere [12]. Several quadrivalent influenza vaccines are now licensed in the US [13-16], including an inactivated vaccine manufactured by GlaxoSmithKline Vaccines in Quebec Canada, which has been shown to be immunogenic with an acceptable safety profile in children and adults [17,18]. This inactivated QIV is provided as a thimerosal-free formulation in a single-dose pre-filled syringe; however, because a multidose presentation is preferred in some clinical settings due to reduced acquisition costs and reduced demand on cold chain storage systems (i.e. multidose vials need less space per dose than prefilled syringes), a multidose presentation containing thimerosal was also being developed [19].

This report describes two, Phase III, non-randomized studies of adults and elderly adults conducted by the same investigator in successive years at a single center in Canada when the recommended strains for a TIV did not change: the first study in 2010–2011 evaluated a TIV in a multidose presentation made by an already licensed manufacturing method wherein the thimerosal preservative was added early in the process (this study was used as an historical control); the second study in 2011–2012, evaluated a candidate formulation of a QIV made by a new process for the production of thimerosal-free vaccine, in which thimerosal preservative was then added during the final manufacturing step to batches to be filled into multidose vials. As the process change could have affected the immunogenicity of the product, we conducted the current study. The rationale for reporting these two studies together is to characterize the immunogenicity, reactogenicity, and safety of the QIV candidate formulation alongside that of the TIV to evaluate the acceptability of the new manufacturing process particularly regarding the European Union Committee for Medicinal Products for Human Use (CHMP) immunogenicity acceptance criteria.

## Methods

Two, Phase III, non-randomized studies were conducted to evaluate the immunogenicity, reactogenicity, and safety of TIV and QIV in adults aged 18–60 years and >60 years. Both studies were conducted at a single center in Canada over consecutive influenza seasons. TIV was assessed during the 2010–2011 season and QIV during the 2011–2012 season; the QIV contained the same strains as the TIV plus a B strain from the alternate lineage. The aim was to describe immunogenicity and safety in younger and older adults, and to observe whether there were any major differences between the TIV and QIV, to evaluate the acceptability of the new manufacturing process (details below) particularly regarding the CHMP immunogenicity acceptance criteria.

Adults aged ≥18 years who were in stable health were eligible to enrol in the studies. Women of child-bearing potential were required to practice reliable contraception for 30 days before and 2 months after vaccination. In both studies, subjects were not eligible for inclusion if they had: received any investigational product 30 days before the study or if this was planned during the study; received any influenza vaccine within 6 months before the study or if this was planned during the study, or participated in a TIV (*Fluviral™* GlaxoSmithKline Vaccines) registration study conducted at the same site in 2009–2010 (exclusion criterion for TIV trial only); received any vaccine within 30 days of the study or if this was planned during the study; acute illness at enrolment or a significant acute or chronic uncontrolled medical or psychiatric illness; had been diagnosed with cancer within 3 years of the study; a history of demyelinating disease; a history of or suspected allergy to any of the vaccine components, or a history of severe adverse reactions to influenza vaccination. Subjects were not eligible for inclusion if they had acute, clinically significant pulmonary, cardiovascular, hepatic or renal functional abnormality, or insulin-dependent diabetes mellitus. Subjects with chronic illness not listed in the exclusion criteria were eligible for inclusion if the illness was stable and controlled.

The study protocols, informed consent and other information requiring pre-approval were reviewed and approved by Institutional Review Boards (Institutional Review Board Services and Optimum Ethics Review Board) in accordance with Canadian regulatory requirements. The studies were conducted in accordance with Good Clinical Practice, the principles of the Declaration of Helsinki, and all regulatory requirements, including Canadian requirements. All subjects gave informed written consent.

### Vaccines

The TIV was an inactivated trivalent, split-virion influenza vaccine (*Fluviral™;* GlaxoSmithKline Vaccines) containing the WHO recommended strains for the 2010–2011 season in the Northern hemisphere: 15 μg hemagglutinin antigen (HA) each of A/California/7/2009 NYMC X-179A (H1N1), A/Victoria/210/2009 NYMC X-187 H3N2 (an A/Perth/16/2009 (H3N2)-like virus), and B/Brisbane/60/2008 (Victoria lineage). The TIV was manufactured with a process containing thimerosal, which was added to the inactivation and splitting steps of the process. During the formulation step, the thimerosal concentration was adjusted to reach a concentration of 100 μg/mL. The TIV was presented in 5.0 mL multidose vials containing 10 doses (0.5 ml) of vaccine.

The QIV was an inactivated quadrivalent, split-virion influenza vaccine (*FluLaval™ Quadrivalent;* GlaxoSmithKline Vaccines) containing the WHO recommended strains for the 2011–2012 season in the Northern hemisphere, which were the same as those for the previous season (as contained in the aforementioned TIV), plus an additional B strain, which was B/Florida/04/2006 (Yamagata lineage). The QIV contained 15 μg HA of each strain. The QIV was manufactured using a new thimerosal free process, which involved the inactivation and splitting of the viruses in absence of thimerosal. Thimerosal was added at the formulation step to reach a concentration of 100 μg/mL as a preservative to those batches to be filled into multidose vials. Exclusively for this study, the thimerosal-containing QIV was presented in pre-filled syringes containing one 0.5 mL dose of vaccine.

Both vaccines were manufactured by GlaxoSmithKline Vaccines, in Quebec, Canada. Subjects received one 0.5 mL dose of TIV or QIV administered intramuscularly in the deltoid region of the non-dominant arm. Each study was open-label.

### Immunogenicity

In the TIV and QIV studies, the primary objective was to assess the humoral immune responses against each vaccine strain 21 days after vaccination in subjects aged 18–60 years and >60 years. Serum samples for antibody testing were obtained on Day 0 before vaccination, and 21 days after vaccination. Antibody responses were assessed using a serum hemagglutination inhibition (HI) assay as previously described [20].

HI antibody responses were described as the anti-log of the arithmetic mean of the log-10 transformed inverse geometric mean titre (GMT), seroprotection rate (SPR; proportion with post-vaccination titer ≥1:40), seroconversion rate (SCR; proportion with pre-vaccination antibody titer <1:10 and with post-vaccination titer of ≥1:40, or pre-vaccination titer of ≥1:10 and a ≥4-fold post-vaccination increase in titer), and mean geometric increase (MGI; geometric mean of the ratio between post-vaccination and pre-vaccination reciprocal HI titers). Subjects with HI antibody titers of ≥1:10 were considered to be seropositive.

### Reactogenicity and safety

The secondary objectives were to assess reactogenicity and safety*.*

Solicited injection site and general adverse events were assessed during the 4 day post-vaccination period. Subjects used diary cards to record solicited injection site symptoms (pain, redness, and swelling/induration) and general symptoms (chest tightness or difficulty in breathing, chills, cough, fatigue, headache, joint pain, muscle pain, red eyes, sore throat/hoarseness/pain on swallowing, swelling of the face, fever), and rated each symptom for severity. Injection-site events were considered to be vaccine-related, and investigators provided causality assessments for general symptoms.

Unsolicited adverse events (AEs) and serious adverse events (SAEs) were assessed from Day 0 to 21 days post-vaccination. All AEs were graded for intensity and recorded according to the Medical Dictionary for Regulatory Activities (MedDRA) classification. Investigators provided causality assessments for unsolicited events.

### Analyses

The sample sizes in the TIV and QIV studies were based on the requirements for annual registration of influenza vaccine in Canada and Europe which stipulate a minimum of 50 subjects are needed in each age stratum.

In both studies, immunogenicity parameters were described with an exact 95% confidence interval (CI). HI immune responses were assessed according to the to the European Union Committee for Medicinal Products for Human Use (CHMP) licensure criteria: to fulfil the criteria the point estimates for SCR needed to be >40%, SPR >70%, and MGI >2.5 in the 18–60 years group, and SCR >30%, SPR >60%, and MGI >2.0 in the >60 years group [21].

Exploratory analyses were conducted to assess immunogenicity in each age stratum (18–60 years and ≥60 years) according to influenza vaccination history. Immune responses were evaluated according to received/did not receive seasonal influenza vaccine during either the preceding three seasons (TIV study; 2009–2010, 2008–2009, 2007–2008) or the preceding season (QIV study; 2010–2011).

The immunogenicity analyses were performed on the per-protocol immunogenicity cohort including all eligible subjects without protocol deviation who had serological data available at a given time point (Day 0, Day 21, or both). The frequency of solicited and unsolicited AEs was tabulated with a 95% CI. Solicited and unsolicited AEs were assessed in all vaccinated subjects (total vaccinated cohort; TVC).

## Results

### Subjects

**Figure 1:**
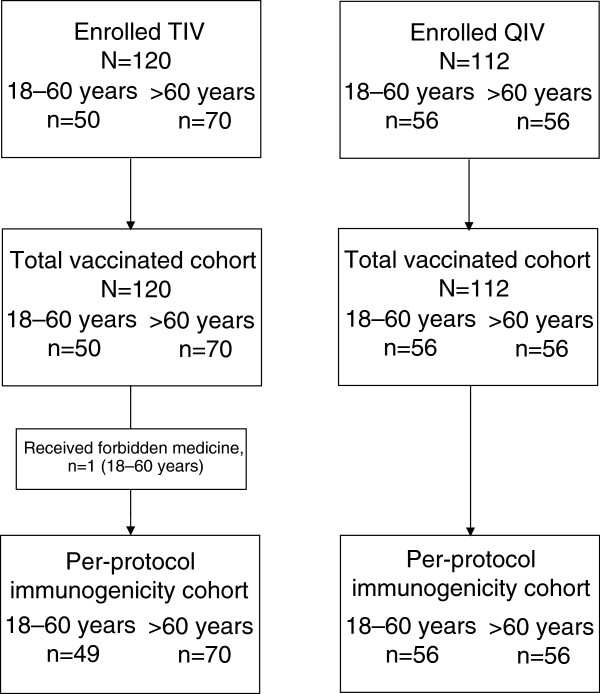
**Subject flow.** QIV, inactivated quadrivalent influenza vaccine. TIV, inactivated trivalent influenza vaccine

Subject flow and analysis groups are shown in Figure 1. In the QIV study, 56 subjects were vaccinated in each age strata, all of which completed the study; the mean age in the 18–60 years group (younger group) was 40.9 years, and in the >60 years group (older group) was 68.6 years (Table 1). In the TIV study, 120 subjects were enrolled and completed; in the TVC at vaccination, there were 50 subjects in the 18–60 years group (younger group), with a mean age of 34.8 years, and 70 subjects aged >60 years group (older group), with a mean age of 66.6 years (Table 1). In the TIV study, in the younger age group, 20 subjects (40%) and in the older group 44 subjects (62.9%) had received seasonal influenza vaccine at least once during the previous 3 seasons.

**Table 1:** Demographic characteristics at baseline in the total vaccinated cohorts

	**TIV**		**QIV**	
	**N = 120**		**N = 112**	
	**18–60 years**	**>60 years**	**18–60 years**	**>60 years**
	**n = 50**	**n = 70**	**n = 56**	**n = 56**
Mean age, years (SD; median; range)	34.8	66.6	40.9	68.6
	(13.72; 28.0; 19.0–59.0)	(4.73; 65.0; 61.0–81.0)	(13.33; 39.5; 22.0–60.0)	(4.72; 67.5; 61.0–82.0)
Male, n (%)	17 (34.0)	31 (44.3)	22 (39.3)	26 (46.4)
Female, n (%)	33 (66.0)	39 (55.7)	34 (60.7)	30 (53.6)
Ethnic origin, n (%)				
White Caucasian/European heritage	47 (94.0)	69 (98.6)	54 (96.4)	56 (100)
Other	3 (6.0)	1 (1.4)	2 (3.6)	0
Seasonal influenza in at least 1 season from previous 3 seasons	20 (40.0)	44 (62.9)	54 (96.4)	54 (96.4)
AS03-adjuvanted pandemic influenza vaccine in the previous (2010–2009) season	26 (52.0)	33 (47.1)	n/a	n/a

TIV, inactivated trivalent influenza vaccine; QIV, inactivated quadrivalent influenza vaccine; SD, standard deviation.

At baseline, GMTs were higher in the QIV study in the younger group (45.3–130.4) and older group (23.2–57.3) compared with the TIV study in the younger (14.1–39.2) and older group (13.0–28.0).

In the TIV study, the first subject was enrolled on 9 July 2010 and the last study visit was on 31 July 2010. In the QIV study, the first subject was enrolled on 30 September 2011 and the last study visit was on 22 October 2011.

### Immunogenicity

#### Quadrivalent vaccine

**Figure 2:**
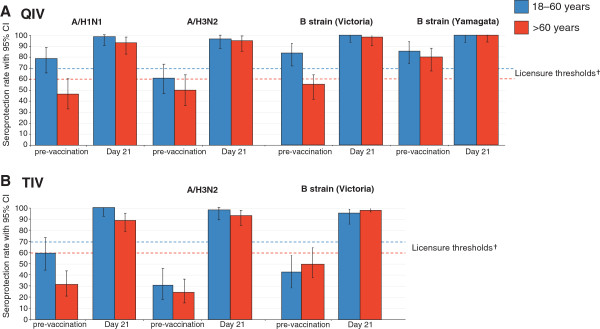
**Seroprotection rates 21 days after QIV (A) or TIV (B) in the per-protocol immunogenicity cohort*.*** QIV, inactivated quadrivalent influenza vaccine; TIV, inactivated trivalent influenza vaccine; Seroprotection rate defined as proportion with post-vaccination titer ≥1:40; European Union Committee for Medicinal Products for Human Use (CHMP) licensure threshold for seroprotection rate: ≥70% in the 18–60 years group, and ≥60% in the >60 years group [17].

**Figure 3:**
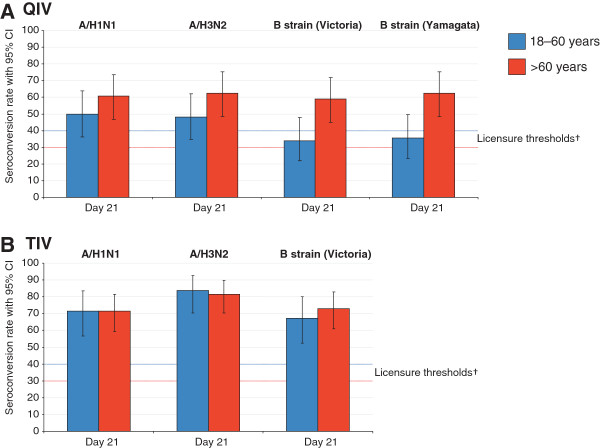
**Seroconversion rates 21 days after QIV (A) or TIV (B) in the per-protocol immunogenicity cohort.** QIV, inactivated quadrivalent influenza vaccine; TIV, inactivated trivalent influenza vaccine; Seroconversion rate defined as the proportion with antibody titer <1:10 at baseline and with post-vaccination titer of ≥1:40, or pre-vaccination titer of ≥1:10 and a ≥4-fold post-vaccination increase in titer; European Union Committee for Medicinal Products for Human Use (CHMP) licensure threshold for seroconversion: >40% in the 18–60 years group, and >30% in the >60 years group [17].

Pre-vaccination seropositivity rates were >87.5% and >71.4% in the younger and older age strata, respectively (Table 2). The QIV was immunogenic in both age strata, and SPRs and MGIs fulfilled CHMP licensure criteria in both age groups against each vaccine strain (Table 2; Figures 2 and 3). SCRs fulfilled licensure criteria for all strains in the older age group, and for the A strains in the younger group. The SCR for B/Yamagata and B/Victoria fell below the licensure threshold in the younger age group at 35.7% and 33.9%, respectively (Figure 3).

**Table 2:** Hemagglutination-inhibition antibody responses after QIV in the per-protocol immunogenicity cohort

**Strain**	**Time point**	**Seropositive**		**GMT**		**MGI**	
		**n (%; 95% CI)**		**value (95% CI)**		**value (95% CI)**	
		**18–60 years**	**>60 years**	**18–60 years**	**>60 years**	**18–60 years**	**>60 years**
		**N = 56**	**N = 56**	**N = 56**	**N = 56**	**N = 56**	**N = 56**
**A/H1N1**	Day 0	49	40	82.0	25.0	–	–
		(87.5; 75.9–94.8)	(71.4; 57.8–82.7)	(54.9–122.5)	(17.3–36.0)		
	Day 21	56	55	392.5	223.5	4.8	8.9
		(100; 93.6–100)	(98.2; 90.4–100)	(305.4–504.4)	(157.0–318.1)	(3.3–7.0)	(5.8–13.7)
**A/H3N2**	Day 0	49	40	45.3	23.2	–	–
		(87.5; 75.9–94.8)	(71.4; 57.8–82.7)	(31.3–65.4)	(16.4–32.8)		
	Day 21	56	55	215.4	160.1	4.8	6.9
		(100; 93.6–100)	(98.2; 90.4–100)	(166.3–278.9)	(115.7–221.4)	(3.5–6.6)	(4.7–10.1)
**B strain (Victoria)**	Day 0	54	52	92.8	38.5	–	–
		(96.4; 87.7–99.6)	(92.9; 82.7–98.0)	(66.5–129.4)	(28.3–52.4)		
	Day 21	56	56	318.0	237.8	3.4	6.2
		(100; 93.6–100)	(100; 93.6–100)	(251.4–402.3)	(179.7–314.9)	(2.5–4.6)	(4.3–8.8)
**B strain (Yamagata)**	Day 0	56	54	130.4	57.3	–	–
		(100; 93.6–100)	(96.4; 87.7–99.6)	(92.8–183.2)	(44.3–74.0)		
	Day 21	56	56	404.8	355.5	3.1	6.2
		(100; 93.6–100)	(100; 93.6–100)	(323.1–507.2)	(263.5–479.5)	(2.4–4.0)	(4.6–8.4)

QIV, inactivated quadrivalent influenza vaccine; Seropositive, titer ≥1:10; GMT, geometric mean titer; MGI, mean geometric increase defined as the geometric mean of the ratio between pre-vaccination and post-vaccination reciprocal titers.

The exploratory analysis of immune response by age and previous seasonal vaccination history showed that previous vaccination reduced immune responses against both B strains in subjects aged 18–60 years. In the younger age stratum, SCRs in subjects who had not been vaccinated against seasonal influenza during the previous year fulfilled licensure criteria for all strains, but in those who had been vaccinated, SCRs did not reach the licensure threshold for A/H1N1 (35.7%), A/H3N1 (32.1%), the Victoria lineage B strain (14.3%) or the Yamagata lineage B strain (21.4%). In the older age stratum, immune responses fulfilled all licensure criteria for each strain regardless of vaccination history (Table 3).

**Table 3:** Hemagglutination-inhibition antibody responses after QIV according to previous seasonal influenza vaccination history in the per-protocol immunogenicity cohort

**Strain**	**Previous vaccination†**	**Timepoint**	**SPR**		**SCR**		**MGI**	
			**n/N (%; 95% CI)**		**n/N (%; 95% CI)**		**N; value (95% CI)**	
			**18–60 years**	**>60 years**	**18–60 years**	**>60 years**	**18–60 years**	**>60 years**
			**N = 28**	**N = 28**	**N = 28**	**N = 28**	**N = 28**	**N = 28**
**A/H1N1**	Yes	Day 0	25/28	16/28	–	–	–	–
			(89.3; 71.8–97.7)	(57.1; 37.2–75.5)				
		Day 21	28/28	25/28	10/28	9/28	28; 3.1	28; 3.5
			(100; 87.7–100)	(89.3; 71.8–97.7)	(35.7; 18.6–55.9)	(32.1; 15.9–52.4)	(2.0–4.9)	(2.3–5.2)
	No	Day 0	19/28	10/28	–	–	–	–
			(67.9; 47.6–84.1)	(35.7; 18.6–55.9)				
		Day 21	27/28	27/28	18/28	25/28	28; 7.4	28; 22.9
			(96.4; 81.7–99.9)	(96.4; 81.7–99.9)	(64.3; 44.1–81.4)	(89.3; 71.8–97.7)	(4.1–13.3)	(12.7–41.2)
**A/H3N2**	Yes	Day 0	21/28	19/28	–	–	–	–
			(75.0; 55.1–89.3)	(67.9; 47.6–84.1)				
		Day 21	27/28	26/28	9/28	13/28	28; 2.7	28; 3.4
			(96.4; 81.7–99.9)	(92.9; 76.5–99.1)	(32.1; 15.9–52.4)	(46.4; 27.5–66.1)	(2.0–3.6)	(2.5–4.7)
	No	Day 0	13/28	9/28	–	–	–	–
			(46.4; 27.5–66.1)	(32.1; 15.9–52.4)				
		Day 21	27/28	27/28	18/28	22/28	28; 8.5	28; 13.8
			(96.4; 81.7–99.9)	(96.4; 81.7–99.9)	(64.3; 44.1–81.4)	(78.6; 59.0–91.7	(5.2–13.9)	(7.4–25.6)
**B strain (Victoria)**	Yes	Day 0	26/28	17/28	–	–	–	–
			(92.9; 76.5–99.1)	(60.7; 40.6–78.5)				
		Day 21	28/28	27/28	4/28	12/28	28; 2.4	28; 3.3
			(100; 87.7–100)	(96.4; 81.7–99.9)	(14.3; 4.0–32.7)	(42.9; 24.5–62.8)	(1.7–3.3)	(2.3–4.9)
	No	Day 0	21/28	14/28	–	–	–	–
			(75.0; 55.1–89.3)	(50.0; 30.6–69.4)				
		Day 21	28/28	28/28	15/28	21/28	28; 4.9	28; 11.5
			(100; 87.7–100)	(100; 87.7–100)	(53.6; 33.9–72.5)	(75.0; 55.1–89.3)	(3.0–7.9)	(6.8–19.2)
**B strain (Yamagata)**	Yes	Day 0	27/28	24/28	–	–	–	–
			(96.4; 81.7–99.9)	(85.7; 67.3–96.0)				
		Day 21	28/28	28/28	6/28	12/28	28; 2.1	28; 4.1
			(100; 87.7–100)	(100; 87.7–100)	(21.4; 8.3–41.0)	(42.9; 24.5–62.8)	(1.6–2.7)	(2.6–6.5)
	No	Day 0	21/28	21/28	–	–	–	–
			(75.0; 55.1–89.3)	(75.0; 55.1–89.3)				
		Day 21	28/28	28/28	14/28	23/28	28; 4.6	28; 9.4
			(100; 87.7–100)	(100; 87.7–100)	(50.0; 30.6–69.4)	(82.1; 63.1–93.9)	(3.1–6.9)	(6.4–13.8)

†Received (yes) or did not receive (no) seasonal influenza vaccine during the preceding season (2010–2011); QIV, inactivated quadrivalent influenza vaccine; n/N, number fulfilling definition of response/total number in group; SCR, seroconversion rate defined as the proportion with antibody titer <1:10 at baseline and with post-vaccination titer of ≥1:40, or pre-vaccination titer of ≥1:10 and a ≥4-fold post-vaccination increase in titer; SPR, seroprotection rate defined as proportion with post-vaccination titer ≥1:40; MGI, mean geometric increase defined as the geometric mean of the ratio between pre-vaccination and post-vaccination reciprocal titers; Immune responses were assessed according to the to the European Union Committee for Medicinal Products for Human Use (CHMP) licensure criteria: to fulfil the criteria the point estimates for SCR needed to be >40%, for SPR >70%, and MGI >2.5 in the 18–60 years group, and SCR >30%, for SPR >60%, and MGI >2.0 in the >60 years group [21].

#### Trivalent vaccine

Pre-vaccination seropositivity rates were higher for the B strain than the A strains, particularly in the >60 years group (Table 4). Post-vaccination GMTs were higher in younger versus older subjects against A/H1N1 (390.1: 95% CI, 293.8–518.1 versus 142.1: 95% CI, 105.1–192.0, respectively), but this was not observed for A/H3N2 or the B strain (Table 4).

**Table 4:** Hemagglutination-inhibition antibody responses after TIV in the per-protocol immunogenicity cohort

**Strain**	**Time point**	**Seropositive**		**GMT**		**MGI**	
		**n (%; 95% CI)**		**value (95% CI)**		**value (95% CI)**	
		**18–60 years**	**>60 years**	**18–60 years**	**>60 years**	**18–60 years**	**>60 years**
		**N = 49**	**N = 70**	**N = 49**	**N = 70**	**N = 49**	**N = 70**
**A/H1N1**	Day 0	35	37	39.2	15.2	–	–
		(71.4; 56.7–83.4)	(52.9; 40.6–64.9)	(24.0–63.8)	(11.1–21.0)		
	Day 21	49	70	390.1	142.1	10.0 (6.3–15.7)	9.3 (6.8–12.8)
		(100; 92.7–100)	(100; 94.9–100)	(293.8–518.1)	(105.1–192.0)		
**A/H3N2**	Day 0	26	38	14.1	13.0	–	–
		(53.1; 38.3–67.5)	(54.3; 41.9–66.3)	(10.1–19.8)	(9.8–17.3)		
	Day 21	49	70	185.6	183.7	13.1 (9.3–18.5)	14.1 (10.2–19.5)
		(100; 92.7–100)	(100; 94.9–100)	(140.9–244.5)	(139.6–241.8)		
**B strain (Victoria)**	Day 0	34	58	22.7	28.0	–	–
		(69.4; 54.6–81.7)	(82.9; 72.0–90.8)	(15.2–34.0)	(21.5–36.5)		
	Day 21	48	69	260.6	248.6	11.5 (7.0–18.7)	8.9 (6.3–12.4)
		(98.0; 89.1–99.9)	(98.6; 92.3–100)	(184.6–368.0)	(185.9–332.3)		

TIV, inactivated trivalent influenza vaccine; Seropositive, titer ≥1:10; GMT, geometric mean titer; MGI, mean geometric increase defined as the geometric mean of the ratio between pre-vaccination and post-vaccination reciprocal titers.

The TIV was immunogenic in both age strata, and SCRs, SPRs, and MGIs fulfilled CHMP licensure criteria in both age groups against each vaccine strain (Figures 2 and 3; Table 5). The exploratory analysis of immune response by age and previous vaccine exposure showed that receipt of seasonal influenza vaccine at least once within the previous three seasons did not have a major effect on subsequent immune responses to the TIV (Table 5). Antibody responses fulfilled licensure criteria in both age strata regardless of previous vaccination history. In the younger group, the SPRs at Day 21 in those with and without previous seasonal influenza vaccination were 100% and 93.1–100%, respectively, and in the older group were 93.2–100% and 80.8–96.2%, respectively. However, there was a trend for lower immune responses in the subjects who had received previous seasonal vaccine based on SCRs and MGIs. In the younger group, the SCRs in those with and without previous seasonal vaccination were 50.0–75.0% and 75.9–89.7%, respectively, and in the older group were 61.4–79.5% and 73.1–92.3%, respectively. In the younger group, the MGIs in those with and without previous vaccination were 5.3–8.9 and 12.6–19.6, respectively. Reduced MGIs were particularly pronounced in the older group between those who were previously vaccinated (5.0–9.9) and those who were not (16.0–25.8).

**Table 5:** Hemagglutination-inhibition antibody responses after TIV according to previous seasonal influenza vaccination history in the per-protocol immunogenicity cohort

**Strain**	**Previous vaccination†**	**Time point**	**SPR**		**SCR**		**MGI**	
			**n/N (%; 95% CI)**		**n/N (%; 95% CI)**		**N; value (95% CI)**	
			**18–60 years**	**>60 years**	**18–60 years**	**>60 years**	**18–60 years**	**>60 years**
**A/H1N1**	Yes	Day 0	13/20	19/44	–	–	–	–
			(65.0; 40.8–84.6)	(43.2; 28.3–59.0)				
		Day 21	20/20	41/44	13/20	31/44	20; 7.1	44; 6.8
			(100; 83.2–100)	(93.2; 81.3–98.6)	(65.0; 40.8–84.6)	(70.5; 54.8–83.2)	(3.5– 14.3)	(4.7–9.8)
	No	Day 0	16/29	3/26	–	–	–	–
			(55.2; 35.7–73.6)	(11.5; 2.4–30.2)				
		Day 21	29/29	21/26	22/29	19/26	29; 12.6	26; 16.0
			(100; 88.1–100)	(80.8; 60.6–93.4)	(75.9; 56.5–89.7)	(73.1; 52.2–88.4)	(6.8– 23.3)	(9.4–27.3)
**A/H3N2**	Yes	Day 0	8/20	15/44	–	–	–	–
			(40.0; 19.1–63.9)	(34.1; 20.5–49.9)				
		Day 21	20/20	43/44	15/20	35/44	20; 8.9	44; 9.9
			(100; 83.2–100)	(97.7; 88.0–99.9)	(75.0; 50.9–91.3)	(79.5; 64.7–90.2)	(4.7– 16.8)	(6.7–14.7)
	No	Day 0	7/29	2/26	–	–	–	–
			(24.1; 10.3–43.5)	(7.7; 0.9–25.1)				
		Day 21	28/29	22/26	26/29	22/26	29; 17.2	26; 25.8
			(96.6; 82.2–99.9)	(84.6; 65.1–95.6)	(89.7; 72.6–97.8)	(84.6; 65.1–95.6)	(11.8–25.1)	(15.7–42.5)
**B strain (Victoria)**	Yes	Day 0	12/20	27/44	–	–		
			(60.0; 36.1–80.9)	(61.4; 45.5–75.6)				
		Day 21	20/20	44/44	10/20	27/44	20; 5.3	44; 5.0
			(100; 83.2–100)	(100; 92.0–100)	(50.0; 27.2–72.8)	(61.4; 45.5–75.6)	(2.7– 10.4)	(3.5–7.0)
	No	Day 0	9/29	8/26	–	–		
			(31.0; 15.3–50.8)	(30.8; 14.3–51.8)				
		Day 21	27/29	25/26	23/29	24/26	29; 19.6	26; 23.9
			(93.1; 77.2–99.2)	(96.2; 80.4–99.9)	(79.3; 60.3–92.0)	(92.3; 74.9–99.1)	(10.3– 37.2)	(14.0–40.6)

†Received (yes) or did not receive (no) seasonal influenza vaccine during the preceding 3 seasons (2009–2010, 2008–2009, 2007–2008); n/N, number fulfilling definition of response/total number in group; TIV, inactivated trivalent influenza vaccine; SCR, seroconversion rate defined as the proportion with antibody titer <1:10 at baseline and with post-vaccination titer of ≥1:40, or pre-vaccination titer of ≥1:10 and a ≥4-fold post-vaccination increase in titer; SPR, seroprotection rate defined as proportion with post-vaccination titer ≥1:40; MGI, mean geometric increase defined as the geometric mean of the ratio between pre-vaccination and post-vaccination reciprocal titers; Immune responses were assessed according to the to the European Union Committee for Medicinal Products for Human Use (CHMP) licensure criteria: to fulfil the criteria the point estimates for SCR needed to be >40%, for SPR >70%, and MGI >2.5 in the 18–60 years group, and SCR >30%, for SPR >60%, and MGI >2.0 in the >60 years group [21].

### Reactogenicity and safety

#### Quadrivalent vaccine

Solicited adverse events during the 4-day post-vaccination period are shown in Table 6. Pain was the most frequent solicited injection site event, and was much more frequent in the younger (41/56; 73.2%) than the older group (19/56; 33.9%). Other solicited injection site events were infrequent in the younger (≤1.8%) and older groups (≤3.6%). There were no Grade 3 injection site events. The most frequent solicited general events in the younger group were muscle pain (21/56; 37.5%), headache (11/56; 19.6%) and fatigue (10/56; 17.9%), and in the older group was muscle pain (6/56; 10.7%). All general AEs reported in both groups were considered by the investigator to be related to vaccination. One subject in the older group reported a Grade 3 event (fatigue).

**Table 6:** Solicited injection site and general adverse events during the 4-day post-vaccination periods in the total vaccinated cohorts

	**TIV**		**QIV**	
	**% (95% CI)**		**% (95% CI)**	
	**18–60 years**	**>60 years**	**18–60 years**	**>60 years**
	**N = 50**	**N = 69**	**N = 56**	**N = 56**
**Injection site events**				
Pain	82.0 (68.6–91.4)	26.1 (16.3–38.1)	73.2 (59.7–84.2)	33.9 (21.8–47.8)
Redness	0.0 (0.0–7.1)	0.0 (0.0–5.2)	1.8 (0.0–9.6)	0.0 (0.0–6.4)
Swelling	4.0 (0.5–13.7)	1.4 (0.0–7.8)	1.8 (0.0–9.6)	3.6 (0.4–12.3)
**General events**				
Chest tightness	0.0 (0.0–7.1)	4.3 (0.9–12.2)	0.0 (0.0–6.4)	3.6 (0.4–12.3)
Chills	4.0 (0.5–13.7)	0.0 (0.0–5.2)	1.8 (0.0–9.6)	8.9 (3.0–19.6)
Cough	4.0 (0.5–13.7)	0.0 (0.0–5.2)	5.4 (1.1–14.9)	7.1 (2.0–17.3)
Fatigue	30.0 (17.9–44.6)	10.1 (4.2–19.8)	17.9 (8.9–30.4)	8.9 (3.0–19.6)
Headache	26.0 (14.6–40.3)	8.7 (3.3–18.0)	19.6 (10.2–32.4)	8.9 (3.0–19.6)
Joint pain at other location	12.0 (4.5–24.3)	4.3 (0.9–12.2)	12.5 (5.2–24.1)	5.4 (1.1–14.9)
Muscle pain	34.0 (21.2–48.8)	8.7 (3.3–18.0)	37.5 (24.9–51.5)	10.7 (4.0–21.9)
Red eyes	8.0 (2.2–19.2)	1.4 (0.0–7.8)	5.4 (1.1–14.9)	0.0 (0.0–6.4)
Sore throat	16.0 (7.2–29.1)	2.9 (0.4–10.1)	12.5 (5.2–24.1)	8.9 (3.0–19.6)
Swelling of the face	2.0 (0.1–10.6)	0.0 (0.0–5.2)	0.0 (0.0–6.4)	0.0 (0.0–6.4)
Increased temperature	2.0 (0.1–10.6)	1.4 (0.0–7.8)	0.0 (0.0–6.4)	0.0 (0.0–6.4)

TIV, inactivated trivalent influenza vaccine; QIV, inactivated quadrivalent influenza vaccine.

During the 21-day post-vaccination period, in the younger group, 13 (23.2%) subjects reported at least 1 unsolicited AE, of which none were Grade 3 or considered to be related to vaccination. In the older group, 12 (21.4%) subjects reported at least 1 unsolicited AE, of which 1 was Grade 3 (arthralgia), and 1 (injection site haemorrhage) was considered to be related to vaccination. There were no SAEs, death or withdrawals due to AE/SAEs during the study.

#### Trivalent vaccine

Solicited adverse events during the 4-day post-vaccination period are shown in Table 6. Pain was the most frequent solicited injection site event, and was much more frequent in the younger (41/50; 82.0%) than the older stratum (18/69; 26.1%). There were no Grade 3 injection site events, and only 3 reports of injection site events other than pain. The most frequent solicited general events in the younger group were muscle pain (17/50; 34.0%), fatigue (15/50; 30.0%) and headaches (13/50; 26.0%), and in the older group were fatigue (7/69; 10.1%), muscle pain (6/69; 8.7%), and headaches (6/69; 8.7%).

During the 21-day post-vaccination period, a total of 16/50 subjects in the younger group (32.0%) repo-rted at least one unsolicited AE, which were most frequently nausea (6.0%) and diarrhoea (6.0%); 8 (16.0%) subjects reported AEs that were considered by the investigator to be related to vaccination, which were abdominal pain, diarrhoea, nausea, fatigue, injection site haematoma, injection site pain, injection site paraesthesia, dizziness, headache, and sedation. During the 21-day post-vaccination period, a total of 17/70 (24.3%) subjects in the older group reported at least one unsolicited AE, which was most frequently headache (4.3%); 3 (4.3%) subjects reported AEs that were considered by the investigator to be related to vaccination, which were nausea, dizziness, and sinus headache.

There were 2 SAEs (Crohn’s disease and nephritis colic), which were both in the younger group and were not considered to be related to vaccination. There were no deaths during the study, and no withdrawals due to AEs/SAEs.

## Discussion

In this analysis of two, Phase III, non-randomized studies, there were no notable differences observed in the immunogenicity, reactogenicity, and safety between a QIV made by a new process wherein the thimerosal preservative was added during the final formulation step, and a TIV made by the already licensed method wherein thimerosal was added early in the manufacturing process (i.e. an historical control). However, our observations are based on a relatively limited sample size, and baseline antibody titers differed between the TIV and QIV populations, particularly regarding the influenza B strains in the older age group.

QIVs developed by GlaxoSmithKline Vaccines that were approved in 2012–2013 in the US and Europe for use in individuals aged ≥3 years are thimerosal-free formulations provided as a single-dose pre-filled syringe. However, GlaxoSmithKline Vaccines has developed a multidose presentation of the QIV manufactured in Quebec, Canada, for use in clinical settings where this product is favored due to its reduced cost of acquisition and cold storage [19]. Multidose vials must contain a preservative to ensure sterility during the period when multiple doses are withdrawn, and thimerosal is the preferred preservative. To harmonize the manufacturing process of the QIV for thimerosal-free and thimerosal-containing presentations, the addition of thimerosal to product destined for filling in multidose vials was delayed to the terminal step of formulation. As this process change could have affected the immunogenicity of the product, we conducted the current study.

We confirmed that the QIV produced by the new manufacturing process provided HI antibody responses against all four vaccine strains which fulfilled all three licensure criteria for immunogenicity 21 days after vaccination in adults aged >60 years. In the 18–60 years group, although all three licensure criteria where fulfilled against the influenza A strains and two of the criteria were fulfilled for the B/Victoria and B/Yamagata strains, seroconversion rates against both influenza B strains fell below the licensure threshold, as would be expected given the particularly high baseline GMTs against the influenza B strains. In the TIV study, HI antibody responses against each vaccine strain fulfilled all three licensure criteria for immunogenicity in both age strata; however, it should be noted that in this study cohort, the baseline HI antibody titers against each vaccine strain were lower than those in the QIV study cohort which confounds a direct comparison.

Various studies have shown that pre-vaccination antibody titers influence HI antibody responses to seasonal influenza vaccines, with lower responses to vaccination reported among subjects with higher pre-vaccination titers derived from natural influenza infection or previous influenza vaccination [22-26]. Moreover, a multivariate analysis based on adults who received a TIV (*Fluarix™*) showed that an age of ≥50 years, previous vaccination within three years, and a pre-vaccination antibody titer of ≥1:40 were predictive of a reduced response to vaccination [27]. To explore this phenomenon, we analyzed antibody responses in subjects according to their previous vaccination history. In the QIV study, responses to all of the QIV strains appeared reduced in younger subjects who had received seasonal influenza vaccine during the previous season compared with those who had not, although this was not observed in older subjects. However, among younger previously vaccinated subjects, GMTs at baseline against the influenza B strains were relatively high. In the TIV study, there was a trend for lower immune responses in the subjects who had received seasonal vaccine at least once during the preceding three seasons, and this trend was most pronounced in the older group.

Overall, the immunogenicity results observed with the thimerosal-containing QIV candidate were consistent with those observed with the thimerosal-containing TIV, and also with the immunogenicity results reported in a previous pivotal Phase III randomized trial of thimerosal-free QIV in adults [18]. In the previous trial, thimerosal-free QIV was immunogenic for all four vaccine strains, eliciting HI antibody responses which compared with TIV were non-inferior for the shared strains and superior for the additional B strain [18].

Pain was the most frequent injection site event, and muscle aches, headache, and fatigue were the most frequent solicited general events in both the QIV and TIV studies, which was consistent with previous reports [18]. However, the incidence of pain was slightly higher in the 18–60 years groups than that previously observed, particularly among TIV recipients. The reason for the relatively high occurrence of injection site pain is unknown, although there were no reports of Grade 3 pain, and all events were mild or moderate in intensity and resolved within four days in the vast majority of subjects.

## Conclusions

In a non-randomized study, we showed that a thimerosal-containing QIV candidate was immunogenic in adults and elderly adults. The reactogenicity profile of the QIV candidate was consistent with the established profile of TIV, and no safety concerns were raised. These results support acceptability of a manufacturing process change in which the thimerosal is added as a preservative at the point at which batches are filled into multidose vials.

## Abbreviations

AE: Adverse events; CHMP: European Union Committee for Medicinal Products for Human Use; CI: Confidence interval; HI: Hemagglutination inhibition; GMT: Geometric mean titre; MedDRA: Medical Dictionary for Regulatory Activities; MGI: Mean geometric increase; SAE: Serious adverse events; SCR: Seroconversion rate; SPR: Seroprotection rate; TIV: Inactivated trivalent influenza vaccines; QIV: Inactivated quadrivalent influenza vaccines; WHO: World Health Organization.

## Competing interest

VK Jain, V Chandrasekaran, L Wang, P Li, A Liu and BL Innis are employees of the GlaxoSmithKline group of companies. VK Jain, P Li, A Liu, L Wang, and BL Innis report ownership of stock options and/or restricted shares.

## Authors’ contribution

All authors participated in the design or implementation or analysis, and interpretation of the study; and the development of this manuscript. All authors had full access to the data and gave final approval before submission. VKJ, LW, AL and BLI managed the study at GlaxoSmithKline Vaccines. VC and PL were responsible for the statistical input.

## Pre-publication history

The pre-publication history for this paper can be accessed here:

http://www.biomedcentral.com/1471-2334/14/133/prepub

## Trademark ownership

*FluLaval™, FluLaval™ Quadrivalent, and Fluviral™* are trademarks of the GlaxoSmithKline group of companies.
